# Supplementary material for: Marine toxin domoic acid alters nitrogen cycling in sediments
Source: Nat Commun. 2023 Nov 30;14:7873. doi: 10.1038/s41467-023-43265-4 (PMC10689436; doi:10.1038/s41467-023-43265-4)
Supplement: Supplementary file 1 — Supplementary Information [file 41467_2023_43265_MOESM1_ESM.pdf]

Supporting Information

Marine Toxin Domoic Acid Alters Nitrogen Cycling in Sediments

Zelong Li [a1], Hao Yue [a1], Jing Wang[a,*], Miaomiao Du [a] , Yuan Jin[b] , Jingfeng Fan[b,*]

[a]*Key Laboratory of Industrial Ecology and Environmental Engineering (Ministry of Education), School of Environmental Science and Technology, Dalian University of Technology, Dalian 116024, P.R. China*

[b]*Marine Ecology Department, National Marine Environmental Monitoring Center, Dalian, 116023, PR China*

[*]Corresponding Author: Jing Wang, Jingfeng Fan

E-mail address: jwang@dlut.edu.cn

[1] Contributed equally to this work.

The Supporting Information has 16 pages including 3 **Texts**, 3 **Tables** and 12 **Figures**.

Table of Contents

Text S1. Detection of DA in sediments 3

Text S2. Rate measurements of potential N transformation rates 4

Text S3. Detection methods for physicochemical properties and heavy metal. 6

Table S1. Relative abundance of N cycling genes of different treatments 7

Table S2. Relative abundance of resistance genes of different treatments 8

Table S3. The information of the co-occurring network nodes in different treatments 9

Figure S1. Changes of DA concentrations in the system 12

Figure S2. Alterations of genes relative abundance in N cycling between $AD_{0.5}$ and AOM groups 13

Figure S3. KEGG functional module analysis of functional pathways associated with N cycling 14

Figure S4. KEGG functional module analysis of functional pathways associated with carbon metabolism 15

Figure S5. Metabolite distribution in different treatments. 16

Figure S6. Marked metabolites in the $AD_{0.5}$ and AOM groups 17

Figure S7. The proportion of TOC and TOC in different treatments 18

Figure S8. The neutral community model fit of microbial community in different treatments 19

Figure S9. Relationships between different N cycling processes and QS genes based on Pearson correlation coefficients 20

Figure S10. Relationships between DA and QS genes based on Pearson correlation coefficients 21

Figure S11. Location of differential metabolites in the metabolic pathway of glycolysis and TCA cycle 22

Figure S12. Biotransformation products of DA 24

Supplementary References 25

Text S1. Detection of DA in sediments.

Sediment DA extractions were conducted according to Sekula-Wood et al.[1]. Briefly, 30 mL of 50% MeOH were added to 5 g sediment wet weight, samples were left on a shaker table at 4 ℃ for 12-24 hours in the dark, centrifuged at 3800 RPM for 10 minutes, and filtered through a 0.2 μm filter. DA was quantified on an LC-MS/MS according to a modified protocol using $^{13}C3$-caffeine as an internal standard. Minimum detection limit for these samples ranged from 0.10-0.15 ng/g depending on the analytical run.

Text S2. Rate measurements of potential N transformation rates.

Sediment slurry incubation experiments, with $^{15}NO_3^-$ ($K^{15}NO_3$, purity ≥ 98.5%, Purchased from aladdin) as a tracer, were conducted after 10 and 25 days incubation time, with exetainer tubes for each treatment replicate (n = 5 per treatment) following Seeley, et al. [2], In brief, exitainer tubes with 2 g of homogenized sediment were helium-purged and dark-incubated overnight to remove residual NO_2^- and NO_3^-. Six replicates of exetainer tubes (12 mL) per sample were amended with 100 mmol $^{15}NO_3^-$ (8 mL) and then incubated under experimental conditions. Both anammox and denitrification activities were stopped by adding saturated zinc chloride (ZnCl) solution after 0, 1, and 2 h of incubations. Time series production of $^{29}N_2$ and $^{30}N_2$ was measured on an isotope ratio mass spectrometer and used to calculate the rate of denitrification and anammox following Jiang, et al. [3].

$^{15}NH_4^+$ was determined by a combination of the ammonium oxidation technique and MIMS analysis (OX/MIMS) [4]. The potential N_2 production and DNRA rate were calculated according to the following equation:

$$R=(K\times V)\div W$$

where R (μmol N kg^{-1} h^{-1}) indicates the measured ^{15}N-based potential N_2 production (R_{N2}) or DNRA rates (R_{DNRA}), K is the slope calculated from the concentration of ^{15}N-N_2 or NH_4^+ versus incubation time, V (L) is the volume of the incubation vial, and W (kg) denotes the dry weight of the sediment. Partitioning of N_2 production and DNRA was expressed as %DNRA (%):

$$\%DNRA=\frac{R_{DNRA}}{R_{N_2}+R_{DNRA}}\times 100\%$$

Potential nitrification rates were determined for 3 replicates from each group, with 2 g of homogenized, centrifuged and supernatant-water-removed sediment [5]. One hundred millilitres of a solution of 1 mM phosphate buffer (0.3 mM KH_2PO_4 + 0.7 mM K_2HPO_4) at pH 7.2 and 0.5 mM $(NH_4)_2SO_4$ was added to 3 g sediments followed by shaking at 150 RPM at 25 °C in aerobic flasks. At $T_t = 0$, a 10 ml aliquot was transferred to a centrifuge tube, five drops of flocculent solution (0.5 M $CaCl_2$ + 0.5 M $MgCl_2$) were added to aliquots before centrifuging for 10 min at 930 g. Five millilitres of clear supernatant were decanted and analysed for NO_3^- + NO_2^- concentration. This procedure was repeated after 18 h of incubation ($T_t = 18$) and PNR were calculated as differences in the concentration of NO_3^- + NO_2^- between $T_t = 18$ and $T_t = 0$. For water samples, quality parameters such as NH_4^+-N, NO_3^--N, and NO_2^--N were determined according to the standard methods [6].

Text S3. Detection methods for physicochemical properties and heavy metal.

Total organic nitrogen and carbon levels were measured with a Vario EL III Element Analyzer (Elementar, Germany) [7]. Sediment pH was measured using a pH meter (Mettler Toledo Fiveplus FE20, Shanghai, China) after the dried sediment was soaked in 2 M KCl with a volume ratio of 2.5.

Concentrations of HCl-extractable Fe^{2+} and Fe^{3+} could be quantified via UV colorimetric assay with 1, 10-phenanthroline method at 530 nm wavelength [8]. Concentrations of other heavy metals in sediments were quantified through (SVDV) ICP-OES (Agilent 5110) after digestion [8].

Table S1. Relative abundance of N cycling genes of different treatments on day 10 and 25 ($AD_{0.5}$ = modified with algal organic matter and 0.5 mg/L domoic acid; AOM = modified with algal organic matter only; CK = without any modification).

Gene	$AD_{0.5}$_10	$AD_{0.5}$_10	$AD_{0.5}$_10	AOM_10	AOM_10	AOM_10	CK_10	CK_10	CK_10
napA	116.565	113.566	111.878	136.641	135.129	131.598	127.56	135.65	125.13
nosZ	369.943	103.192	94.689	370.378	170.541	168.793	473.64	103.284	60.211
nirK	26.491	23.683	21.644	32.956	25.271	25.954	25.442	24.508	23.573
nirS	32.632	37.659	30.113	38.069	48.929	50.521	38.823	37.323	39.433
norB	150.071	150.902	138.975	192.376	199.739	187.078	204.19	191.372	189.67
narG	31.265	38.608	15.767	27.593	31.636	28.714	51.982	18.155	21.721
nrfA	6.195	6.872	7.049	8.554	6.105	6.632	1.577	0.285	0
amoC	18.3	16.63	21.487	11.851	11.811	12.797	10.104	13.628	11.431
hao	2.385	1.859	2.62	0.12	1.21	0	1.23	1.24	1.2
nxrA	31.265	38.608	15.767	7.593	7.636	8.714	51.982	18.155	21.721
hzsB	1.2123	7.543	1.310	3.508	7.282	8.454	17.310	13.658	17.690
ureC	26.271	28.954	29.543	23.686	26.562	23.697	17.581	16.445	18.819
nifH	17.67	16.664	12.658	34.803	54.67	55.959	37.197	37.385	34.542

Gene	$AD_{0.5}$_25	$AD_{0.5}$_25	$AD_{0.5}$_25	AOM_25	AOM_25	AOM_25	CK_25	CK_25	CK_25
napA	54.696	73.23	38.116	69.188	72.358	81.304	23.542	54.613	57.887
nosZ	350.637	180.99	79.987	230.184	339.855	339.865	472.85	245.79	248.99
nirK	6.288	8.515	7.474	18.965	21.052	15.188	16.644	40.567	19.21
nirS	10.04	14.551	8.94	16.759	19.164	11.207	16.306	37.774	63.273
norB	78.097	132.536	59.593	89.678	165.962	157.612	73.064	141.45	179.8
narG	33.051	22.721	41.771	38.223	41.102	45.294	49.185	32.123	62.457
nrfA	36.454	24.911	24.206	4.255	5.614	5.805	3.308	1.31	5.576
amoC	88.104	73.725	64.129	32.972	32.978	36.877	12.145	77.498	59.905
hao	0	2.847	0	3.22	2.32	1.11	0	5.722	5.739
nxrA	33.051	22.721	41.771	8.223	11.102	15.294	49.185	32.123	62.457
hzsB	1.3123	1.2565	3.632	20.773	28.384	56.77	1.594	4.370	6.249
ureC	23.425	23.21	22.652	21.548	22.961	23.967	13.815	17.595	15.008
nifH	2.272	5.024	0.433	11.152	0.638	14.136	0	9.495	11.3

Table S2. Relative abundance of resistance genes of different treatments on day 10 and 25 ($AD_{0.5}$ = modified with algal organic matter and 0.5 mg/L domoic acid; AOM = modified with algal organic matter only; CK = without any modification).

Gene	$AD_{0.5}$_10	$AD_{0.5}$_10	$AD_{0.5}$_10	AOM_10	AOM_10	AOM_10	CK_10	CK_10	CK_10
FliL	5.761	5.848	4.694	2.636	0.376	1.205	1.808	0	1.847
FlhF	5.761	5.848	4.694	5.913	3.191	4.029	1.808	0	1.847
CheB	14.551	21.832	21.006	10.154	6.567	5.859	4.246	1.837	2.895
CheD	1.219	7.694	8.834	4.977	1.371	1.889	0.693	0.963	2.944
CheR	9.778	7.427	9.808	6.73	6.429	6.914	7.835	4.318	0
CheZ	4.028	5.168	2.989	5.698	2.053	1.199	1.098	0	0.583
RimM	172.699	152.563	180.542	98.027	91.294	89.323	106.125	122.306	116.724
sbcD	7.159	7.473	6.995	5.831	2.5	3.732	0.776	1.313	0.32
LptA	10.855	10.364	12.165	7.049	7.401	5.813	0	0	1.032

Gene	$AD_{0.5}$_25	$AD_{0.5}$_25	$AD_{0.5}$_25	AOM_25	AOM_25	AOM_25	CK_25	CK_25	CK_25
FliL	174.256	137.128	195.33	120.658	31.976	34.656	272.159	77.723	97.537
FlhF	9.915	9.528	8.635	2.153	3.351	4.861	0.89	0.621	0.756
CheB	36.392	66.599	101.54	10.033	5.692	7.778	16.715	18.252	12.298
CheD	27.774	41.8	93.746	7.955	14.101	14.557	16.313	22.804	9.21
CheR	64.82	82.516	142.731	5.957	5.052	4.415	15.782	14.062	15.455
CheZ	5.414	5.636	4.405	1.098	1.056	0	2.154	2.272	0.692
RimM	49.092	74.742	114.669	32.205	38.023	34.116	36.1	46.558	55.166
sbcD	20.776	18.777	14.382	2.368	5.783	3.575	0.151	0.639	5.7
LptA	122.783	62.29	64.316	10.849	29.663	28.593	6.877	53.078	52.965

Table S3. The information of the co-occurring network nodes in different treatments ($AD_{0.5}$ = modified with algal organic matter and 0.5 mg/L domoic acid; AOM = modified with algal organic matter only; CK = without any modification).

Number	$AD_{0.5}$	AOM	CK
1	Methanosarcinaceae	Methanomicrobiaceae	Methanomicrobiaceae
2	Nitrosopumilaceae	Methanosarcinaceae	Methanotrichaceae
3	Iamiaceae	Nitrosopumilaceae	Nitrosopumilaceae
4	Actinomycetaceae	Iamiaceae	Iamiaceae
5	Corynebacteriaceae	Ilumatobacteraceae	Actinomycetaceae
6	Dietziaceae	Bifidobacteriaceae	Bifidobacteriaceae
7	Geodermatophilaceae	Mycobacteriaceae	Corynebacteriaceae
8	Brevibacteriaceae	Nocardiaceae	Gordoniaceae
9	Intrasporangiaceae	Geodermatophilaceae	Mycobacteriaceae
10	Ornithinimicrobiaceae	Beutenbergiaceae	Nocardiaceae
11	Promicromonosporaceae	Cellulomonadaceae	Geodermatophilaceae
12	Micromonosporaceae	Dermatophilaceae	Brevibacteriaceae
13	Nakamurellaceae	Micrococcaceae	Cellulomonadaceae
14	Nocardioidaceae	Ornithinimicrobiaceae	Micromonosporaceae
15	Propionibacteriaceae	Propionibacteriaceae	Nocardioidaceae
16	Streptomycetaceae	Nocardiopsaceae	Propionibacteriaceae
17	Coriobacteriaceae	Euzebyaceae	Atopobiaceae
18	Bacteroidaceae	Miltoncostaeaceae	Eggerthellaceae
19	Prevotellaceae	Bacteroidaceae	Bacteroidaceae
20	Marinifilaceae	Dysgonomonadaceae	Dysgonomonadaceae
21	Prolixibacteraceae	Prevotellaceae	Rikenellaceae
22	Bernardetiaceae	Marinifilaceae	Tannerellaceae
23	Cyclobacteriaceae	Prolixibacteraceae	Marinifilaceae
24	Spirosomaceae	Cyclobacteriaceae	Chitinophagaceae
25	Crocinitomicaceae	Spirosomaceae	Bernardetiaceae
26	Schleiferiaceae	Cryomorphaceae	Cyclobacteriaceae
27	Weeksellaceae	Flavobacteriaceae	Cytophagaceae
28	Balneolaceae	Schleiferiaceae	Marivirgaceae
29	Bacillaceae	Weeksellaceae	Spirosomaceae
30	Staphylococcaceae	Balneolaceae	Crocinitomicaceae
31	Enterococcaceae	Anaerolineaceae	Flavobacteriaceae
32	Lactobacillaceae	Phototrophicaceae	Schleiferiaceae
33	Streptococcaceae	Calditerrivibrionaceae	Weeksellaceae
34	Clostridiaceae	Bacillaceae	Balneolaceae
35	Lachnospiraceae	Staphylococcaceae	Chlorobiaceae
36	Oscillospiraceae	Carnobacteriaceae	Planococcaceae
37	Peptostreptococcaceae	Lactobacillaceae	Staphylococcaceae
38	Turicibacteraceae	Oscillospiraceae	Enterococcaceae
39	Kiritimatiellaceae	Peptostreptococcaceae	Lactobacillaceae
40	Nitrospinaceae	Erysipelotrichaceae	Streptococcaceae
41	Nitrospiraceae	Turicibacteraceae	Lachnospiraceae
42	Lacipirellulaceae	Fusobacteriaceae	Syntrophomonadaceae

43	Pirellulaceae	Nitrospiraceae	Erysipelotrichaceae
44	Aurantimonadaceae	Isosphaeraceae	Turicibacteraceae
45	Beijerinckiaceae	Pirellulaceae	Nitrospinaceae
46	Brucellaceae	Planctomycetaceae	Nitrospiraceae
47	Chelatococcaceae	Ahrensiaceae	Candidatus
48	Devosiaceae	Beijerinckiaceae	Lacipirellulaceae
49	Hyphomicrobiaceae	Chelatococcaceae	Pirellulaceae
50	Methylobacteriaceae	Devosiaceae	Planctomycetaceae
51	Parvibaculaceae	Hyphomicrobiaceae	Acidithiobacillaceae
52	Phyllobacteriaceae	Nitrobacteraceae	Caulobacteraceae
53	Rhizobiaceae	Parvibaculaceae	Ahrensiaceae
54	Rhodobiaceae	Phyllobacteriaceae	Beijerinckiaceae
55	Stappiaceae	Rhizobiaceae	Breoghaniaceae
56	Hyphomonadaceae	Rhodobiaceae	Chelatococcaceae
57	Maricaulaceae	Xanthobacteraceae	Devosiaceae
58	Parvularculaceae	Rhodobacteraceae	Hyphomicrobiaceae
59	Rhodobacteraceae	Roseobacteraceae	Methylobacteriaceae
60	Roseobacteraceae	Sphingomonadaceae	Methylocystaceae
61	Rhodospirillaceae	Burkholderiaceae	Nitrobacteraceae
62	Erythrobacteraceae	Comamonadaceae	Rhizobiaceae
63	Sphingomonadaceae	Oxalobacteraceae	Rhodobiaceae
64	Alcaligenaceae	Nitrosomonadaceae	Hyphomonadaceae
65	Burkholderiaceae	Sterolibacteriaceae	Maricaulaceae
66	Comamonadaceae	Sulfuricellaceae	Parvularculaceae
67	Oxalobacteraceae	Zoogloeaceae	Rhodobacteraceae
68	Chromobacteriaceae	Desulfobacteraceae	Roseobacteraceae
69	Nitrosomonadaceae	Desulfocapsaceae	Thalassospiraceae
70	Sulfuricellaceae	Desulfuromonadaceae	Sphingomonadaceae
71	Rhodocyclaceae	Geobacteraceae	Alcaligenaceae
72	Zoogloeaceae	Syntrophotaleaceae	Burkholderiaceae
73	Desulfobulbaceae	Archangiaceae	Comamonadaceae
74	Desulfocapsaceae	Myxococcaceae	Oxalobacteraceae
75	Desulfovibrionaceae	Arcobacteraceae	Chromobacteriaceae
76	Geobacteraceae	Thiovulaceae	Gallionellaceae
77	Archangiaceae	Acidiferrobacteraceae	Nitrosomonadaceae
78	Kofleriaceae	Alteromonadaceae	Sterolibacteriaceae
79	Myxococcaceae	Colwelliaceae	Sulfuricellaceae
80	Sulfurovaceae	Pseudoalteromonadaceae	Zoogloeaceae
81	Thiovulaceae	Shewanellaceae	Desulfobacteraceae
82	Acidiferrobacteraceae	Cellvibrionaceae	Desulfobulbaceae
83	Alteromonadaceae	Spongiibacteraceae	Desulfocapsaceae
84	Idiomarinaceae	Granulosicoccaceae	Desulfohalobiaceae
85	Halieaceae	Morganellaceae	Desulfovibrionaceae
86	Chromatiaceae	Yersiniaceae	Geobacteraceae
87	Ectothiorhodospiraceae	Kangiellaceae	Syntrophotaleaceae
88	Granulosicoccaceae	Moraxellaceae	Archangiaceae
89	Woeseiaceae	Alcanivoracaceae	Kofleriaceae
90	Yersiniaceae	Oceanospirillaceae	Myxococcaceae
91	Alcanivoracaceae	Marinobacteraceae	Sandaracinaceae
92	Halomonadaceae	Pseudomonadaceae	Arcobacteraceae

93	Oceanospirillaceae	Piscirickettsiaceae	Sulfurospirillaceae
94	Pseudomonadaceae	Thiotrichaceae	Sulfurovaceae
95	Piscirickettsiaceae	Vibrionaceae	Thiovulaceae
96	Thiotrichaceae	Xanthomonadaceae	Acidiferrobacteraceae
97	Vibrionaceae	Akkermansiaceae	Aeromonadaceae
98	Xanthomonadaceae	Aspergillaceae	Alteromonadaceae
99	Acholeplasmataceae	Sclerotiniaceae	Idiomarinaceae
100	Pleosporaceae	Debaryomycetaceae	Pseudoalteromonadaceae
101	Debaryomycetaceae	Metschnikowiaceae	Cellvibrionaceae
102	Clavicipitaceae	Saccharomycetaceae	Spongiibacteraceae
103	Phycomycetaceae	Glomerellaceae	Chromatiaceae
104	Myoviridae	Cryptococcaceae	Ectothiorhodospiraceae
105	Podoviridae	Myoviridae	Granulosicoccaceae
106		Podoviridae	Morganellaceae
107		Siphoviridae	Yersiniaceae
108		Methanobacteriaceae	Kangiellaceae
109			Moraxellaceae
110			Alcanivoracaceae
111			Oceanospirillaceae
112			Marinobacteraceae
113			Pseudomonadaceae
114			Piscirickettsiaceae
115			Vibrionaceae
116			Xanthomonadaceae
117			Candidatus
118			Botryosphaeriaceae
119			Pleosporaceae
120			Herpotrichiellaceae
121			Trichocomaceae
122			Sclerotiniaceae
123			Debaryomycetaceae
124			Saccharomycopsidaceae
125			Glomerellaceae
126			Plectosphaerellaceae
127			Clavicipitaceae
128			Hypocreaceae
129			Ophiocordycipitaceae
130			Xylonaceae
131			Suillaceae
132			Cryptococcaceae
133			Phycomycetaceae
134			Autographiviridae
135			Myoviridae
136			Podoviridae
137			Methanobacteriaceae

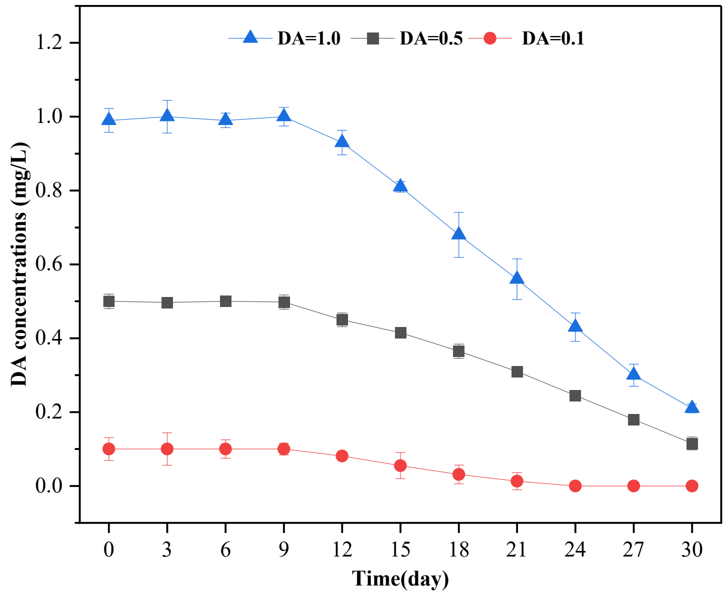

Figure S1. Changes of DA (domoic acid) concentrations in the system.

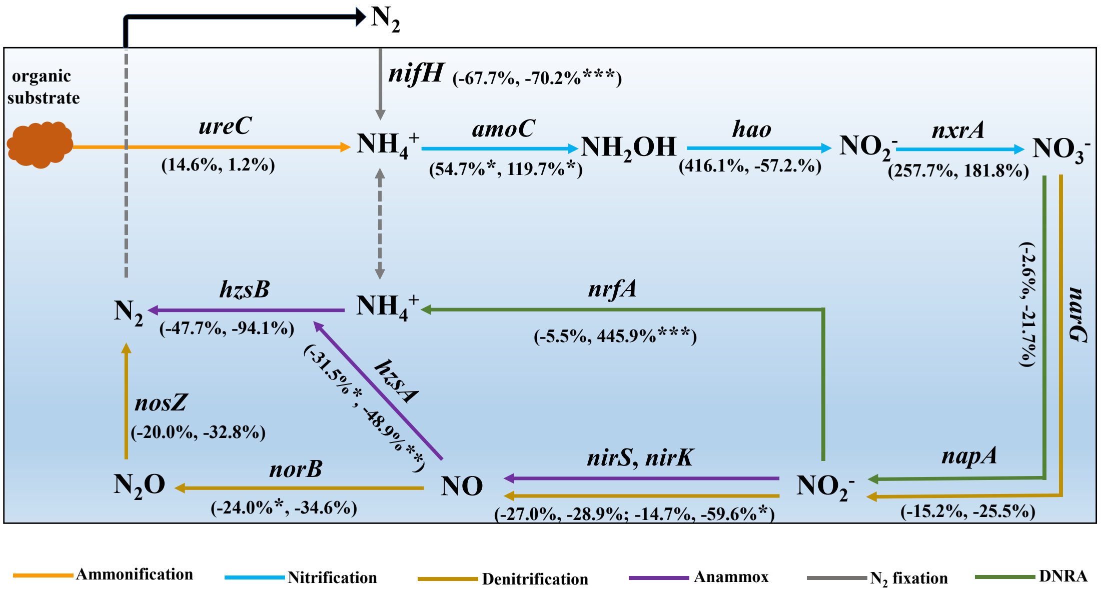

Figure S2. Alterations of genes relative abundance in nitrogen cycling between $AD_{0.5}$ and AOM groups. The percentage changes of each gene in brackets represents the change in $AD_{0.5}$ group relative to the AOM group on day 10 and day 25, respectively (100%× ((mean value in $AD_{0.5}$ group/mean value in AOM group) – 1)). Nitrogen cycling: ammoniation, nitrification, denitrification, Anammox, N_2 fixation, DNRA (dissimilatory nitrate reduction to ammonium). $AD_{0.5}$ = modified with algal organic matter and 0.5 mg/L domoic acid, AOM = modified with algal organic matter only. *$p \leq 0.05$，**$p \leq 0.01$，***$p \leq 0.001$.

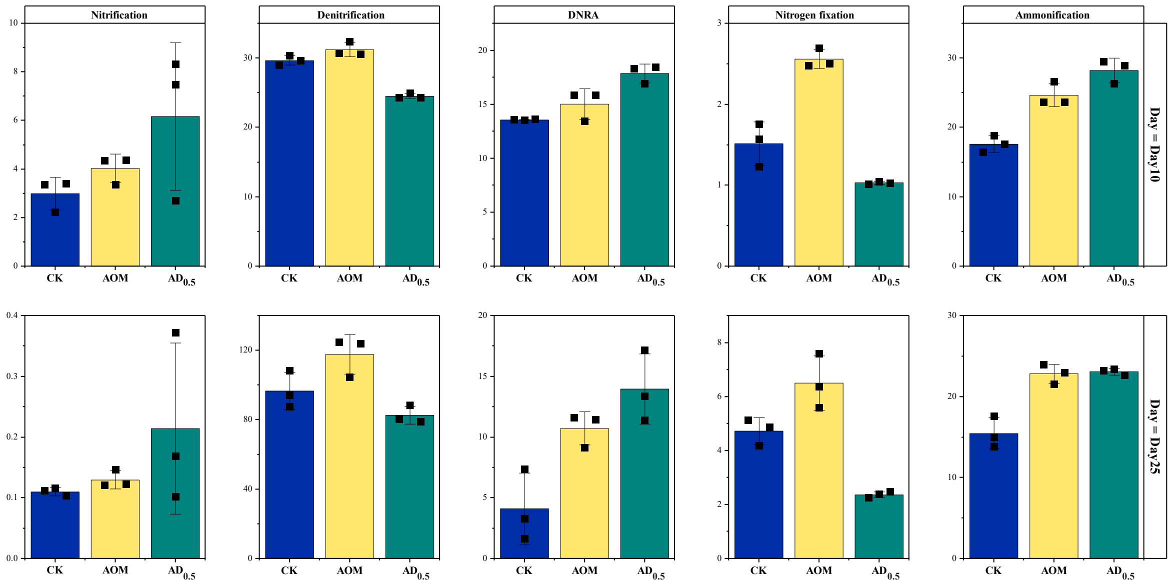

Figure S3. KEGG functional module analysis of functional pathways associated with nitrogen cycling of different treatments on day 10 and 25, (n = 3 biological replicates). Nitrogen cycling: nitrification, denitrification, nitrogen fixation, ammonification, DNRA (dissimilatory nitrate reduction to ammonium). CK = without any modification, AOM = modified with algal organic matter only, $AD_{0.5}$ = modified with algal organic matter and 0.5 mg/L domoic acid. Error bars represent mean ± standard deviation.

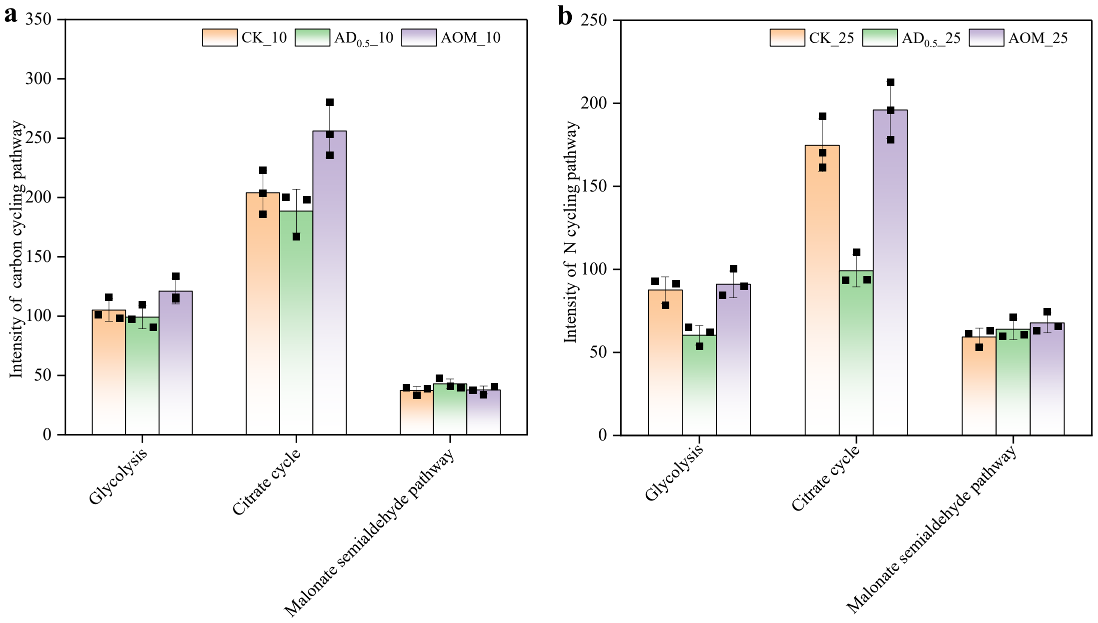

Figure S4. KEGG functional module analysis of glycolysis, malonate semialdehyde pathway and TCA cycle (tricarboxylic acid cycle) of different treatments on day 10 (a) and 25 (b), (n = 3 biological replicates). CK = without any modification, $AD_{0.5}$ = modified with algal organic matter and 0.5 mg/L domoic acid, AOM = modified with algal organic matter only. Error bars represent mean ±standard deviation.

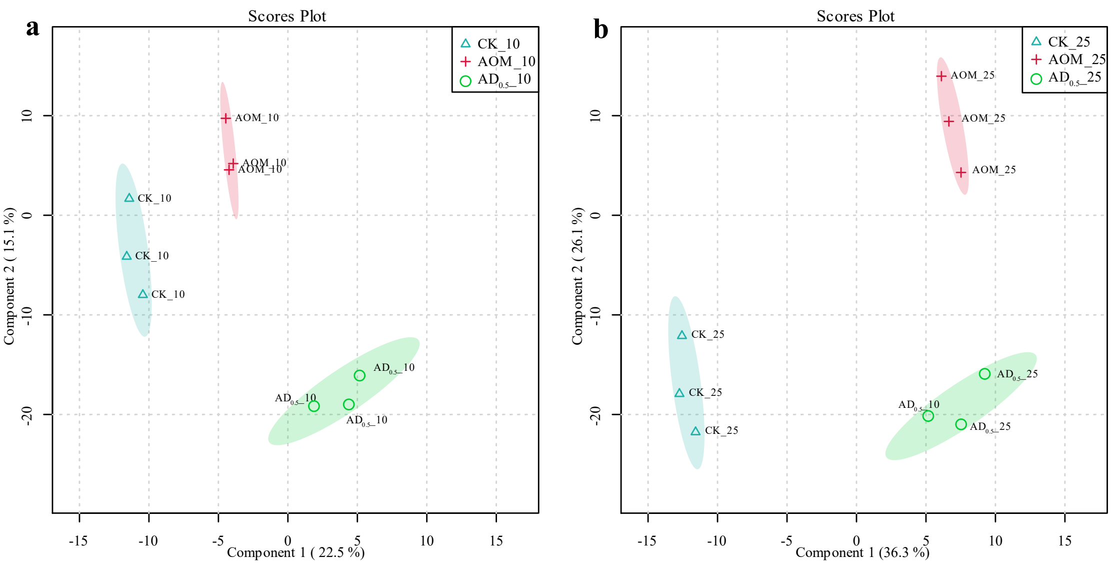

Figure S5. Metabolite distribution in different treatments on day 10 (a) and 25 (b) as determined by PLS-DA (Partial Least Squares Discrimination Analysis). Different groups are marked with different colors, and the area marked by the ellipse is the 95% confidence region of the sample points. CK = without any modification, AOM = modified with algal organic matter only, $AD_{0.5}$ = modified with algal organic matter and 0.5 mg/L domoic acid.

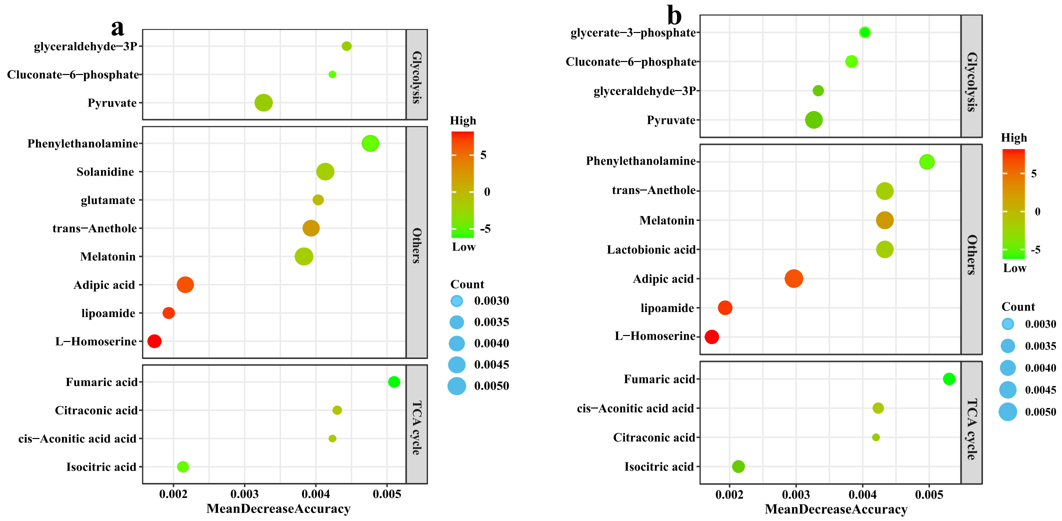

Figure S6. Marked metabolites in the $AD_{0.5}$ and AOM groups on day 10 (a) and 25 (b), respectively. The horizontal coordinate of the graph is "Mean Decrease Accuracy", as indicated by the size of the Counts, measuring the importance of metabolites in the "random forest". A higher value indicates a greater importance of the metabolite. The heat map shows the content of the 15 metabolites in the two groups. TCA cycle = tricarboxylic acid cycle. $AD_{0.5}$ = modified with algal organic matter and 0.5 mg/L domoic acid, AOM = modified with algal organic matter only.

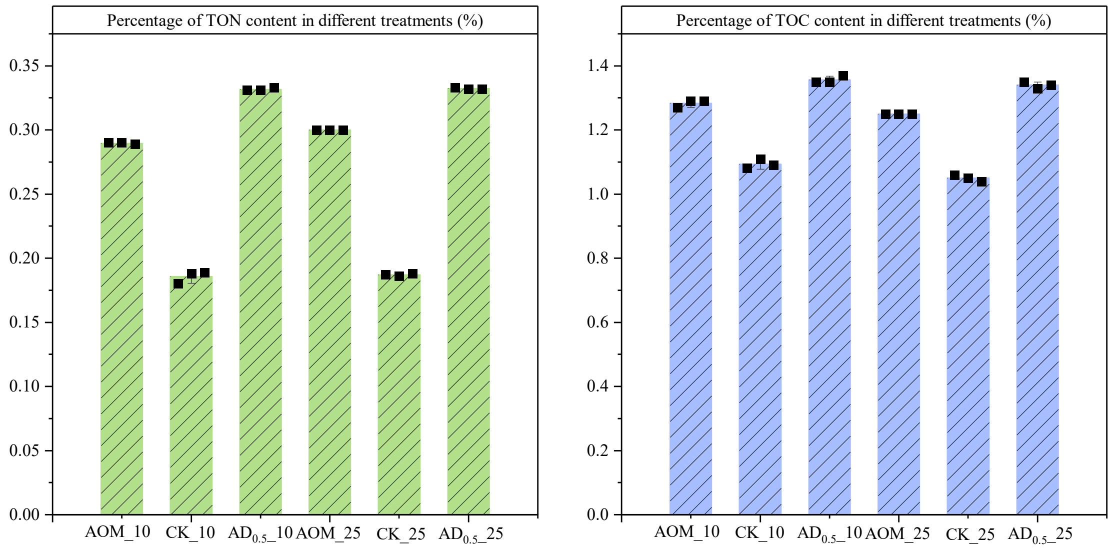

Figure S7. The proportion of TON (total organic nitrogen) and TOC (total organic carbon) on day 10 and in different treatments, (n = 3 biological replicates). CK = without any modification, AOM = modified with algal organic matter only, $AD_{0.5}$ = modified with algal organic matter and 0.5 mg/L domoic acid. Error bars represent mean ± standard deviation.

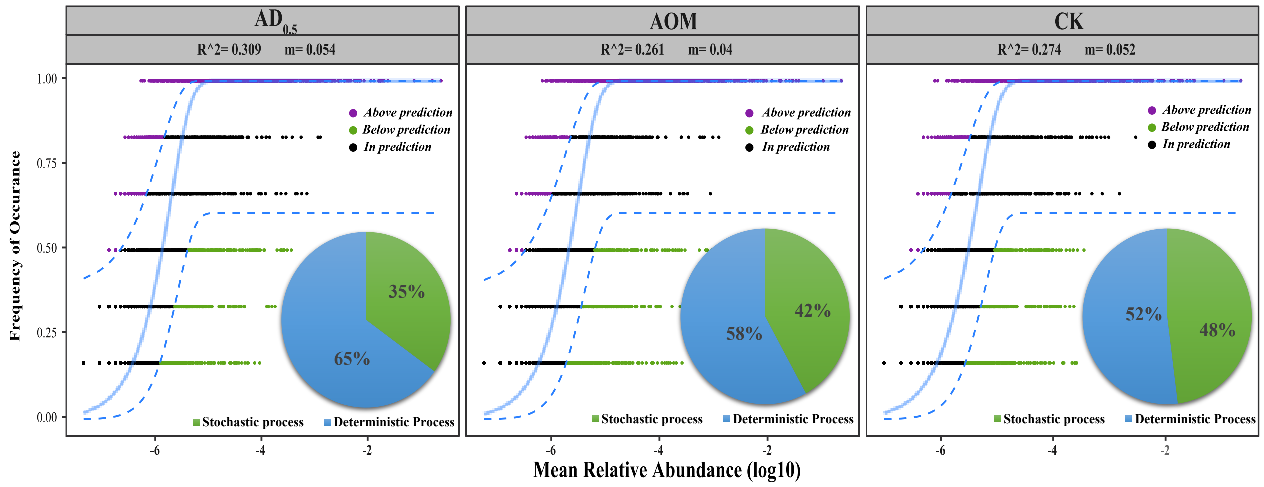

Figure S8. The neutral community model fit of microbial community in different treatments, where R^2 represents the goodness of fit of the model and m represents the migration rate of the species. The percentage in the pie chart represents the stochastic and deterministic ratio in different treatments based on NST (Normalized Stochasticity Ratio) index during assembly. CK = without any modification, AOM = modified with algal organic matter only, $AD_{0.5}$ = modified with algal organic matter and 0.5 mg/L domoic acid.

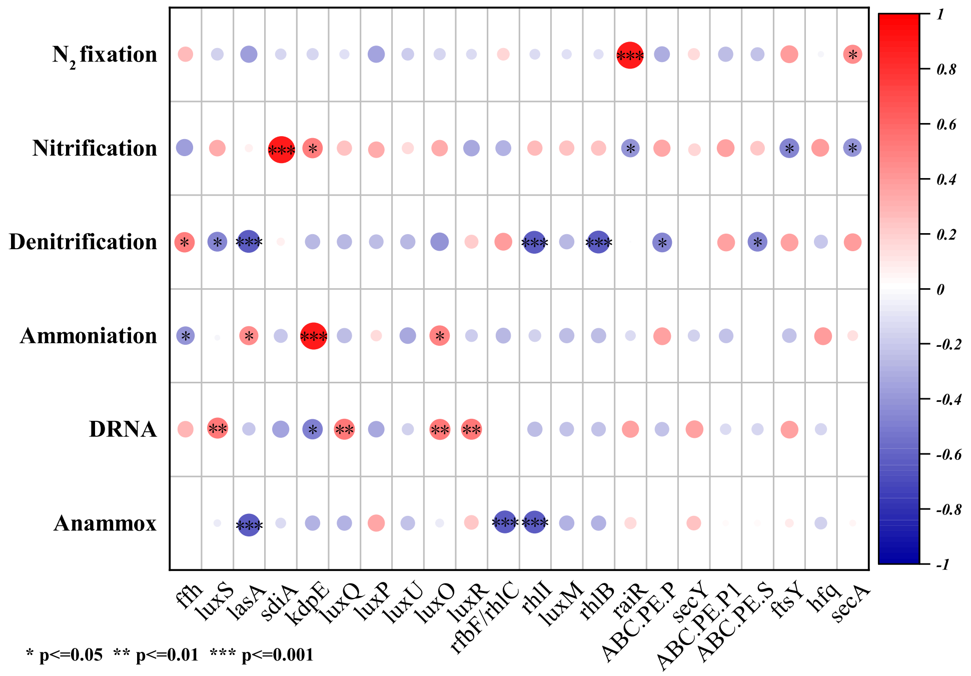

Figure S9. Relationships between different nitrogen cycling and quorum sensing genes based on Pearson correlation coefficients. Nitrogen cycling: nitrification, denitrification, nitrogen fixation, Anammox, ammonification, DNRA (dissimilatory nitrate reduction to ammonium).

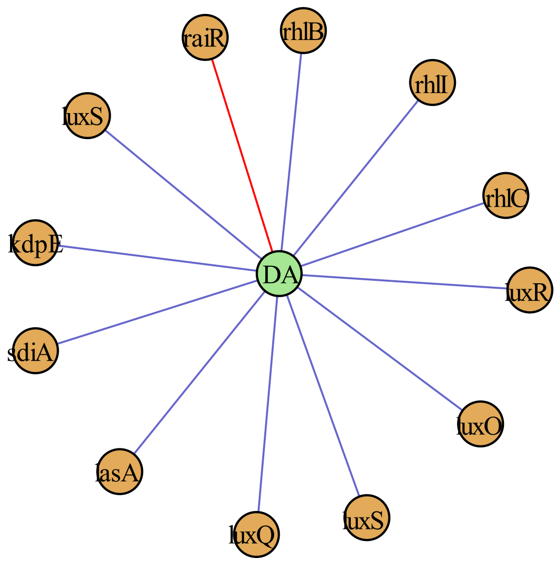

Figure S10. Relationships between DA (domoic acid) and QS (quorum sensing) genes based on Pearson correlation coefficients. The red link reflects positive covariation between DA and QS genes, whereas a blue link reflects negative covariation.

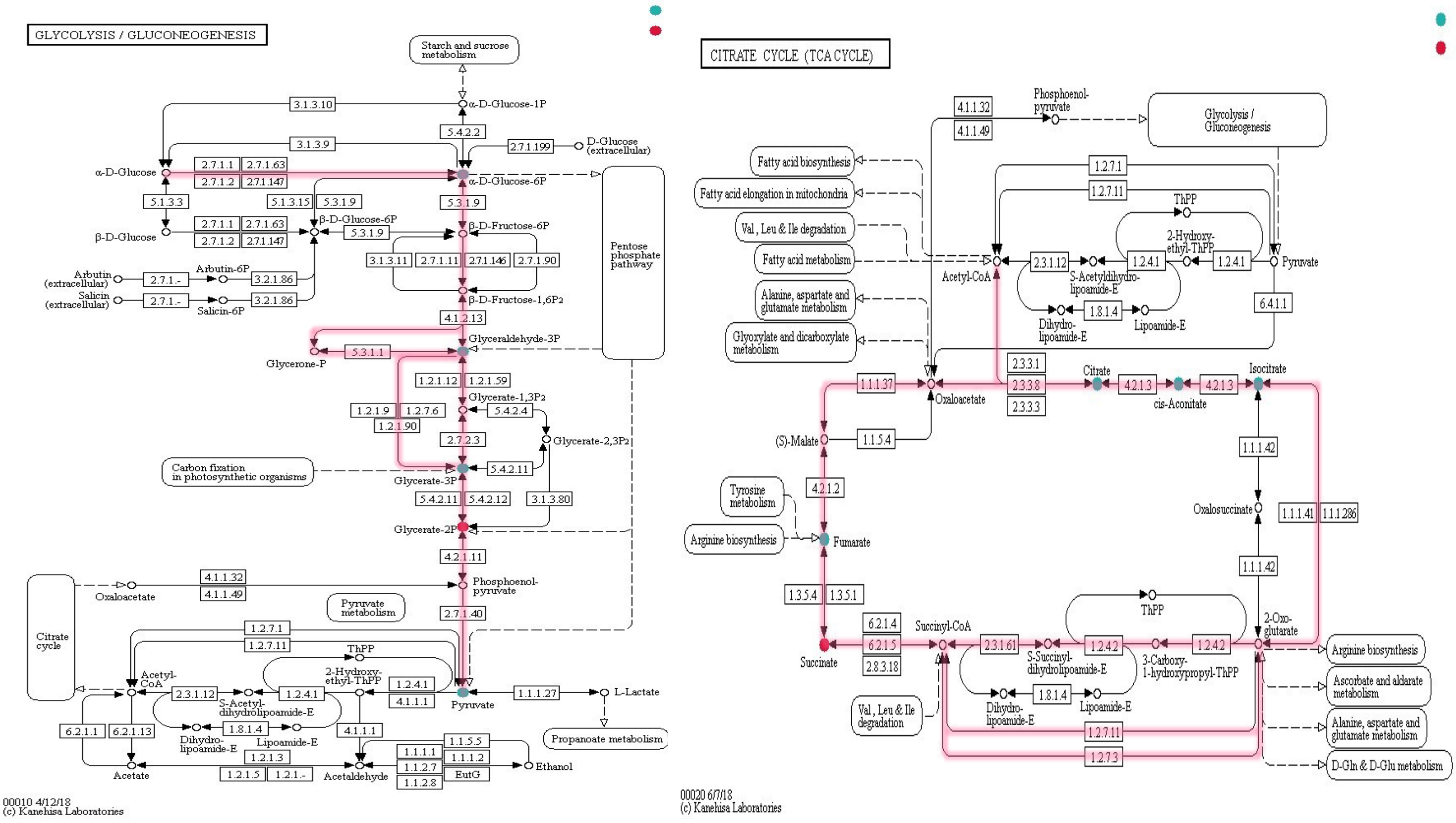

Figure S11. Location of differential metabolites in the metabolic pathway of glycolysis and TCA cycle (tricarboxylic acid cycle), with red representing upregulated metabolites and green representing downregulated metabolites.

Name: P_{345} (m/z=345)

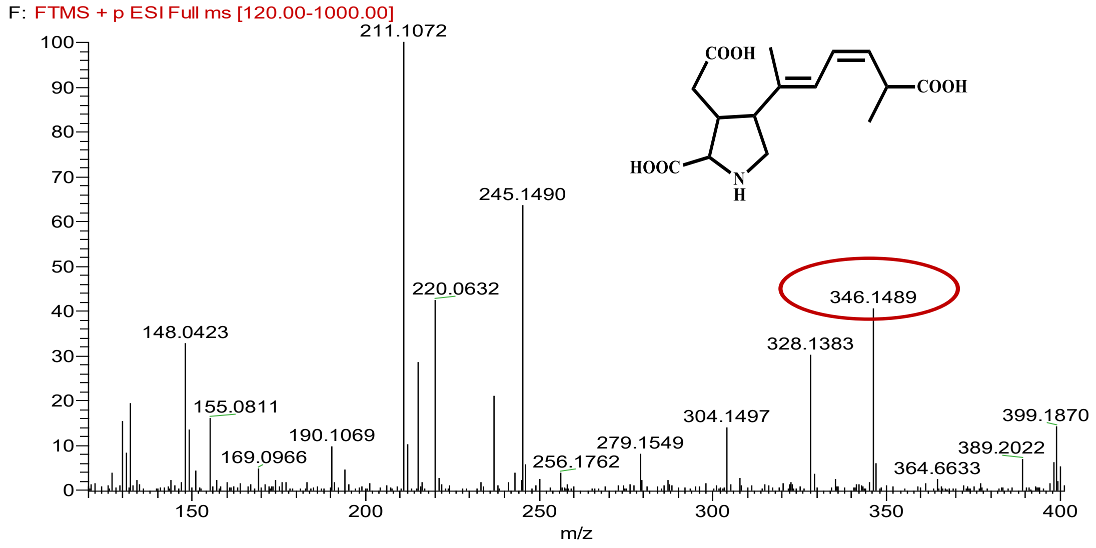

Name: P_{309} (m/z=309)

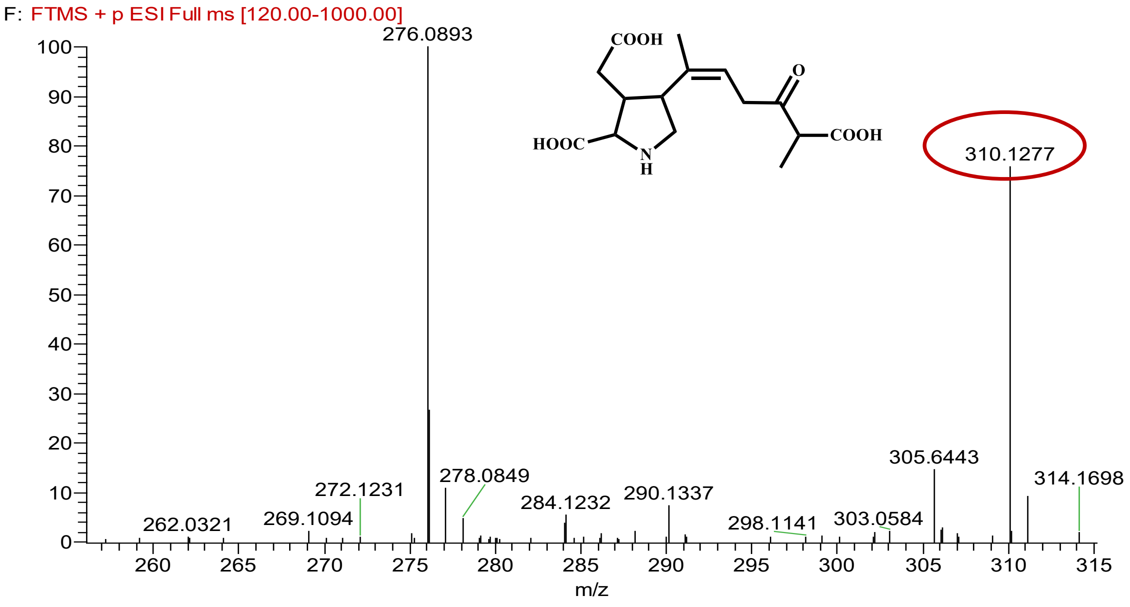

Name: P_{271} (*m/z*=271)

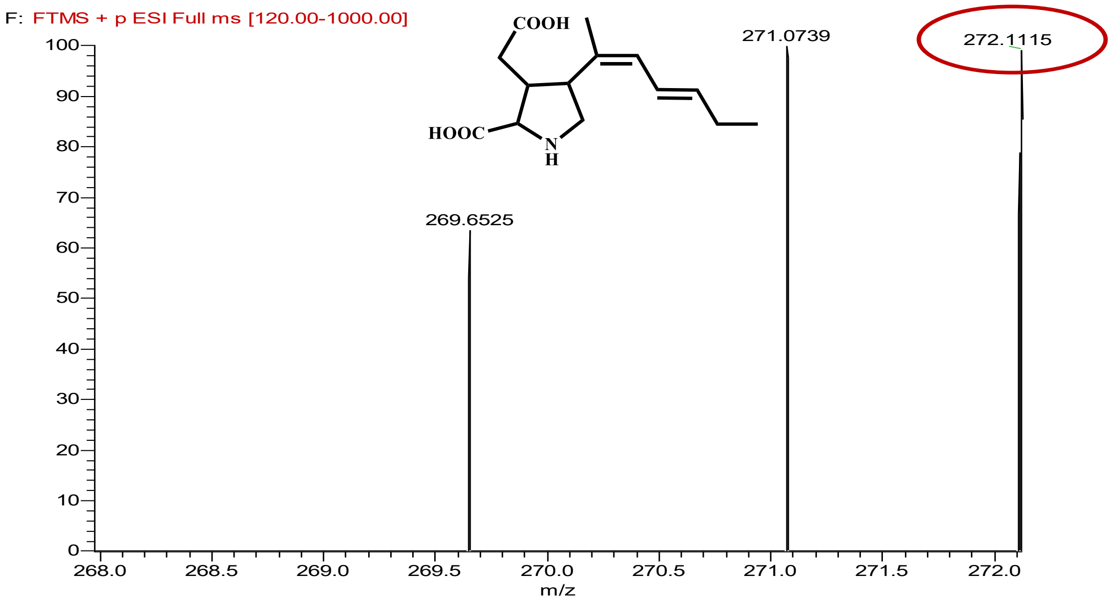

Figure S12. Biotransformation products of domoic acid.

Supplementary References

1 Sekula-Wood, E. *et al.* Pseudo-nitzschia and domoic acid fluxes in Santa Barbara Basin (CA) from 1993 to 2008. *Harmful Algae* **10**, 567-575 (2011).

2 Seeley, M. E., Song, B., Passie, R. & Hale, R. C. Microplastics affect sedimentary microbial communities and nitrogen cycling. *Nature Communications* **11**, doi:10.1038/s41467-020-16235-3 (2020).

3 Jiang, X. Y. *et al.* Role of algal accumulations on the partitioning between N-2 production and dissimilatory nitrate reduction to ammonium in eutrophic lakes. *Water Res* **183**, doi:ARTN 116075
10.1016/j.watres.2020.116075 (2020).

4 Yin, G. Y., Hou, L. J., Liu, M., Liu, Z. F. & Gardner, W. S. A Novel Membrane Inlet Mass Spectrometer Method to Measure (NH4+)-N-15 for Isotope-Enrichment Experiments in Aquatic Ecosystems. *Environ Sci Technol* **48**, 9555-9562, doi:10.1021/es501261s (2014).

5 Petersen, D. G. *et al.* Abundance of microbial genes associated with nitrogen cycling as indices of biogeochemical process rates across a vegetation gradient in Alaska. *Environ Microbiol* **14**, 993-1008, doi:10.1111/j.1462-2920.2011.02679.x (2012).

6 Rice, E. W., Baird, R. B., Eaton, A. D. & Clesceri, L. S. *Standard methods for the examination of water and wastewater*. Vol. 10 (American public health association Washington, DC, 2012).

7 Han, Y. *et al.* Fishmeal Application Induces Antibiotic Resistance Gene Propagation in Mariculture Sediment. *Environ Sci Technol* **51**, 10850-10860, doi:10.1021/acs.est.7b02875 (2017).

8 Gao, S. M. *et al.* Patterns and ecological drivers of viral communities in acid mine drainage sediments across Southern China. *Nature Communications* **13**, doi:ARTN 2389
10.1038/s41467-022-30049-5 (2022).
