# Supplementary material for: Marine toxin domoic acid alters nitrogen cycling in sediments
Source: Nat Commun. 2023 Nov 30;14:7873. doi: 10.1038/s41467-023-43265-4 (PMC10689436; doi:10.1038/s41467-023-43265-4)
Supplement: Supplementary file 3 — Description of Additional Supplementary Files [file 41467_2023_43265_MOESM3_ESM.pdf]

Description of Additional Supplementary Files:

Supplementary Data 1: The impact of different treatments on the relative abundance of carbon metabolism genes.
