# Supplementary material for: Marine toxin domoic acid alters nitrogen cycling in sediments
Source: Nat Commun. 2023 Nov 30;14:7873. doi: 10.1038/s41467-023-43265-4 (PMC10689436; doi:10.1038/s41467-023-43265-4)
Supplement: Supplementary file 5 — Reporting Summary [file 41467_2023_43265_MOESM5_ESM.pdf]

nature portfolio

Corresponding author(s): Jing Wang;Jingfeng Fan

Last updated by author(s): May 10, 2023

Reporting Summary

Nature Portfolio wishes to improve the reproducibility of the work that we publish. This form provides structure for consistency and transparency in reporting. For further information on Nature Portfolio policies, see our Editorial Policies and the Editorial Policy Checklist.

Statistics

For all statistical analyses, confirm that the following items are present in the figure legend, table legend, main text, or Methods section.

n/a	Confirmed	
☐	☒	The exact sample size (*n*) for each experimental group/condition, given as a discrete number and unit of measurement
☐	☒	A statement on whether measurements were taken from distinct samples or whether the same sample was measured repeatedly
☐	☒	The statistical test(s) used AND whether they are one- or two-sided<br>*Only common tests should be described solely by name; describe more complex techniques in the Methods section.*
☐	☒	A description of all covariates tested
☐	☒	A description of any assumptions or corrections, such as tests of normality and adjustment for multiple comparisons
☐	☒	A full description of the statistical parameters including central tendency (e.g. means) or other basic estimates (e.g. regression coefficient) AND variation (e.g. standard deviation) or associated estimates of uncertainty (e.g. confidence intervals)
☐	☒	For null hypothesis testing, the test statistic (e.g. *F*, *t*, *r*) with confidence intervals, effect sizes, degrees of freedom and *P* value noted<br>*Give P values as exact values whenever suitable.*
☒	☐	For Bayesian analysis, information on the choice of priors and Markov chain Monte Carlo settings
☒	☐	For hierarchical and complex designs, identification of the appropriate level for tests and full reporting of outcomes
☐	☒	Estimates of effect sizes (e.g. Cohen's *d*, Pearson's *r*), indicating how they were calculated

Our web collection on statistics for biologists contains articles on many of the points above.

Software and code

Policy information about availability of computer code

Data collection	MEGAHIT software (v1.2.9)<br>EggNOG database (v5.0, http://eggnog5.embl.de/#/app/home)<br>KEGG database (v102.0, https://www.kegg.jp/kegg/)<br>GO database (released in 2023-05-16 https://geneontology.org/)<br>COG database (v2020, https://www.ncbi.nlm.nih.gov/COG/)<br>CAZy database (v2020-05, http://www.cazy.org/)<br>Diamond software (v0.8.22, v2.0.14)<br>NCyc database (https://github.com/qichao1984/NCyc)<br>NCBI NR protein database (https://ftp.ncbi.nih.gov/blast/db)<br>BASTA software (v1.4)<br>Kraken2 (v2.0.7-beta)<br>NT nucleic acid database and RefSeq whole genome database of NCBI (https://ftp.ncbi.nih.gov/blast/db))<br>Bracken software(v2.0)<br>mzCloud software(https://www.mzcloud.org/)
Data analysis	Statistical analyses were performed using R (v4.2.1)<br>plspm package (v0.5.0)<br>stats4 package (v4.3.0)<br>minpack.lm package (v1.2-3)

Hmisc package (v5.1-0)
NST package (v3.1.10)
igraph package (v1.4.3)
Gephi software(v0.9.6)
Cytoscape software (v3.9.1)
Originpro software (v2023)
custom scripts (https://doi.org/10.5281/zenodo.8431607)

For manuscripts utilizing custom algorithms or software that are central to the research but not yet described in published literature, software must be made available to editors and reviewers. We strongly encourage code deposition in a community repository (e.g. GitHub). See the Nature Portfolio guidelines for submitting code & software for further information.

Data

Policy information about availability of data

All manuscripts must include a data availability statement. This statement should provide the following information, where applicable:

- Accession codes, unique identifiers, or web links for publicly available datasets
- A description of any restrictions on data availability
- For clinical datasets or third party data, please ensure that the statement adheres to our policy

The macrogenomic data generated in this study have been deposited in National Ge-nomics Data Center under accession code PRJNA912632 (https://www.ncbi.nlm.nih.gov/bioproject/PRJNA912632). The databases used to obtain the annotation information are as follows: EggNOG database (http://eggnog5.embl.de/#/app/home) to obtain the KEGG (https://www.kegg.jp/kegg/), GO (https://geneontology.org/), COG (https://www.ncbi.nlm.nih.gov/COG/), and CAZy database (http://www.cazy.org/). NT nucleic acid database and RefSeq whole genome database of NCBI can be found at https://ftp.ncbi.nih.gov/blast/db. NCyc database is available at https://github.com/qichao1984/NCyc. The authors declare that the remainder of data that support the findings of this study are available within the article and source data file (https://doi.org/10.6084/m9.figshare.22817291) 58.

Research involving human participants, their data, or biological material

Policy information about studies with human participants or human data. See also policy information about sex, gender (identity/presentation), and sexual orientation and race, ethnicity and racism.

Reporting on sex and gender	This work does not involve human participants, their data, or biological material
Reporting on race, ethnicity, or other socially relevant groupings	This work does not involve human participants, their data, or biological material
Population characteristics	This work does not involve human participants, their data, or biological material
Recruitment	This work does not involve human participants, their data, or biological material
Ethics oversight	This work does not involve human participants, their data, or biological material

Note that full information on the approval of the study protocol must also be provided in the manuscript.

Field-specific reporting

Please select the one below that is the best fit for your research. If you are not sure, read the appropriate sections before making your selection.

☐ Life sciences ☐ Behavioural & social sciences ☒ Ecological, evolutionary & environmental sciences

For a reference copy of the document with all sections, see nature.com/documents/nr-reporting-summary-flat.pdf

Ecological, evolutionary & environmental sciences study design

All studies must disclose on these points even when the disclosure is negative.

Study description	We investigated the single- and interaction-effects of the marine toxin domoic acid on sediment microbial communities. For the first time, it was determined that domoic acid can act as a stressor, inducing alterations in the sediment nitrogen cycling.
Research sample	Since this work intends to investigate the effect of the marine toxin domoic acid on coastal sediments nitrogen cycling, we chose coastal seawater and sediments as samples.
Sampling strategy	The sediments and seawater were collected from the top 2 cm of the Heishijiao coast, located along the Yellow Sea, Dalian, China at low tide in April 2022. We intend to use offshore seawater and sediments to establish the microcosms, so we take as many samples as possible to make sufficient preparation for the experiment.
Data collection	Our samples have been entrusted to a third-party company for testing, and the experimental data has been uploaded to the public database.

Timing and spatial scale	The experiment began on April 22, 2022. On the 10th day (May 2, 2022) and the 25th day (May 17, 2022) of the experiment, three microcosms from each treatment were randomly selected for sampling and sequencing, and relevant data were collected.
Data exclusions	no data were excluded from the analyses
Reproducibility	All samples in this experiment were set with three biological replicates.
Randomization	The samples are randomly grouped.
Blinding	The conditions and parameters of this experiment are not easily susceptible to subjective influences, as we have rigorously controlled the pollutant concentrations and the handling of sediment samples. Furthermore, environmental experiments may be challenging to conduct under single-blind or double-blind conditions, as they necessitate direct interventions, and pursuing blinding measures may render the experimental design impractical.

Did the study involve field work? ☐ Yes ☒ No

Reporting for specific materials, systems and methods

We require information from authors about some types of materials, experimental systems and methods used in many studies. Here, indicate whether each material, system or method listed is relevant to your study. If you are not sure if a list item applies to your research, read the appropriate section before selecting a response.

Materials & experimental systems

n/a	Involved in the study
☒	☐ Antibodies
☒	☐ Eukaryotic cell lines
☒	☐ Palaeontology and archaeology
☒	☐ Animals and other organisms
☒	☐ Clinical data
☒	☐ Dual use research of concern
☒	☐ Plants

Methods

n/a	Involved in the study
☒	☐ ChIP-seq
☒	☐ Flow cytometry
☒	☐ MRI-based neuroimaging
